# Correlation of histopathological findings and magnetic resonance imaging in the spine of patients with ankylosing spondylitis

**DOI:** 10.1186/ar2035

**Published:** 2006-08-22

**Authors:** Heiner Appel, Christoph Loddenkemper, Zarko Grozdanovic, Harald Ebhardt, Marc Dreimann, Axel Hempfing, Harald Stein, Peter Metz-Stavenhagen, Martin Rudwaleit, Joachim Sieper

**Affiliations:** 1 Charité Berlin, Campus Benjamin Franklin, Department of Gastroenterology, Infectiology and Rheumatology, Hindenburgdamm 30, 12200 Berlin, Germany; 2 Charité Berlin, Campus Benjamin Franklin, Department of Pathology, Hindenburgdamm 30, 12200 Berlin, Germany; 3 Charite Berlin, Campus Benjamin Franklin, Department of Radiology, Hindenburgdamm 30, 12200 Berlin, Germany; 4 Werner-Wicker-Klinik, Department II, Center for Spine Surgery, Im Kreuzfeld 4, 34537 Bad Wildungen, Germany

## Abstract

Ankylosing spondylitis (AS) is a chronic inflammatory disease which affects primarily the sacroiliac joints and the spine. In patients with active disease, magnetic resonance imaging (MRI) of the spine shows areas of bone marrow edema, the histopathological equivalent of which is unknown. In this study we correlate inflammation in the spine of patients with AS as revealed by histological examination with bone marrow edema as detected by MRI. We have compared the histopathological findings of zygapophyseal joints from 8 patients with AS (age: 30 to 64, disease duration 7 to 33 years) undergoing spinal surgery with findings in MRI. For histopathological analysis, we quantified infiltrates of CD3+, CD4+ and CD8+ T cells as well as CD20+ B cells immunohistochemically. Bone marrow edema was evaluated in hematoxylin and eosin stained sections and quantified as the percentage of the bone marrow area involved. All patients with AS showed interstitial mononuclear cell infiltrates and various degrees of bone marrow edema (range from 10% to 60%) in histopathological analysis. However, in only three of eight patients histopathological inflammation and edema in the zygapophyseal joints correlated with bone marrow edema in zygapophyseal joints of the lumbar spine as detected by MRI. Interestingly, two of these patients showed the highest histological score for bone marrow edema (60%). This first study correlating histopathological changes in the spine of patients with AS with findings in MRI scans suggests that a substantial degree of bone marrow inflammation and edema is necessary to be detected by MRI.

## Introduction

The prevalence of ankylosing spondylitis (AS) within Caucasians has been estimated to be between 0.2% and 0.8% [1,2]. About 20% of these patients with AS have bridging syndesmophytes which result in restricted movement of the spine as a consequence of active inflammation in spinal joints and adjacent structures [3]. It has been suggested that the involvement of zygapophyseal joints is important in the restriction of spinal mobility [3-5]. This is supported by computed tomography observations, which displayed a significant correlation between changes in the zygapophyseal joints and restriction of spinal mobility [6,7].

Acute inflammation in the spine associated with AS, as detected by magnetic resonance imaging (MRI), has been described in the intervertebral disc, in the vertebra, enthesis of interspinal ligaments, costovertebral joints and zygapophyseal joints [8]. However, systematic MRI of zygapophyseal joints has not been undertaken so far.

As a consequence, a correlation of histopathological analysis of the spine and inflammation as visualized by MRI has also not previously been done. In the sacroiliac joints of patients with AS, a correlation of MRI findings and histopathological evaluations from needle biopsies out of the same sacroiliac joints was reported, but without description and quantification of histological edema [9].

We have recently reported the first systematic histological study of zygapophyseal joints in patients with AS [10]. In the present study, we first examined whether inflammation in the spine of patients with AS, as detected by histopathology, can be correlated with bone marrow edema detected by MRI and consequently whether a negative MRI excludes active inflammation of the spine in patients with AS.

## Materials and methods

### Patients

Zygapophyseal joints were obtained from eight patients with AS (mean age 45 (range 30 to 59); five men, three women). This study includes patients from a larger group for which histopathological observations from the zygapophyseal joints were reported recently [10]. In brief, all eight patients had severe kyphosis and were completely ankylosed in the lumbar spine. The mean disease duration of all eight patients was 22.5 years (range 7 to 33 years). Seven of eight patients reported symptoms of nocturnal back pain before surgery. Histologically detectable edema and cellular infiltrates were also searched for in control samples taken from autopsies of 10 patients without AS who died from cardiovascular diseases and had no history of rheumatic diseases.

**Figure 1:**
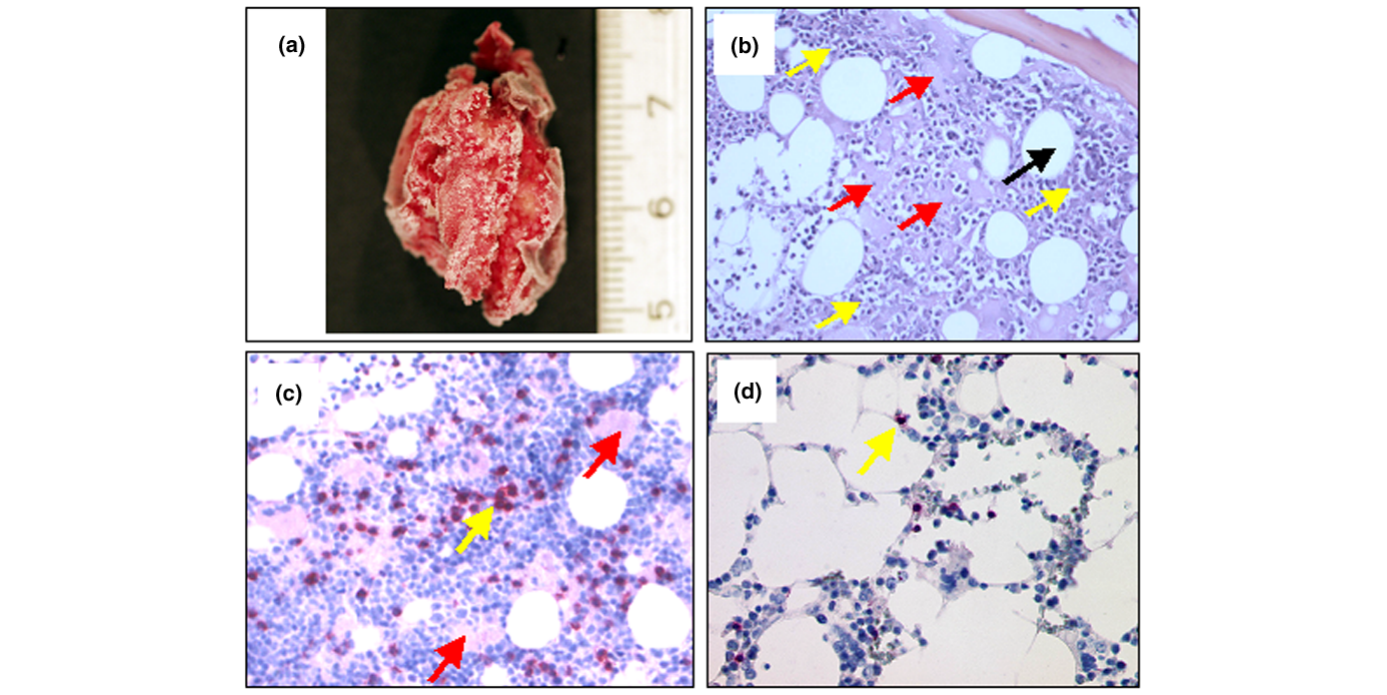
Macroscopic and microscopic assessment of zygapophyseal joints. **(a) **Macroscopic picture of a zygapophyseal joint from a patient with ankylosing spondylitis (AS). **(b) **Hematoxylin and eosin staining of a zygapophyseal joint from an AS patient: weakly eosinophilic fluid accumulation in the bone marrow interstitium (red arrows) neighboring dense interstitial infiltrates of mononuclear cells (yellow arrows). The black arrow indicates fat vacuoles. **(c) **Immunohistochemical analysis of CD3^+ ^T cells in the zygapophyseal joint of an AS patient: dense formations of CD3^+ ^T cells (red stain, yellow arrow) neighboring bone marrow edema (red arrow). **(d) **Immunohistochemical analysis of CD3^+ ^T cells in a zygapophyseal joint of a non-AS control with only few loosely distributed CD3^+ ^T cells in the bone marrow without edema.

Surgery was performed as reported in more detail previously [10]: we obtained at least one zygapophyseal joint (Figure 1a) from the lumbar spine of each patient with AS. For all eight patients with AS, preoperative MR images of the lumbar spine and the thoracic spine were available for comparative analysis.

Permission for this study was given by the local ethic committee of the Charité Berlin, Campus Benjamin Franklin. All patients gave permission for histopathological analysis of the obtained material.

### Histological assessment

The histopathological assessment was performed as described recently [11]. In brief, the zygapophyseal joints were cut into thin slices (3 to 4 mm) with a saw and fixed in 4% buffered formalin. After decalcification with EDTA, sections 4 to 6 μm thick were prepared and stained with hematoxylin and eosin for the evaluation of bone marrow edema. Bone marrow edema was characterized by the accumulation of eosinophilic fluid in the bone marrow interstitium in between univacuolar fat cells, resembling the morphological findings described in the bone marrow of patients with severe malnutrition [12], during the first couple of weeks following stem cell transplantation [13] or after cytoreductive therapy in chronic idiopathic myelofibrosis [14] (Figure 1b; red arrows, edema; black arrow, vacuoles of fat cells). The relative amount of edema, calculated as the percentage of the total bone marrow area, was quantified as described in an earlier study in which bone marrow edema was assessed for a comparative analysis of magnetic resonance (MR) images and histopathology in osteoarthritis [15]. The sections were also examined by polarized light after staining with Congo red to rule out the presence of amyloid mimicking extracellular edema.

The evaluation of all sections was performed by an experienced pathologist (CL) who was blinded for patients, controls, and the MRI results. Sections were examined with a microscope allowing light and immune fluorescence microscopy (BX60; Olympus, Hamburg, Germany). Pictures were taken with a digital camera (Color View II; Soft Imaging System GmbH, Münster, Germany) and were further analyzed by Analysis^® ^software (Soft Imaging System).

### Immunohistochemistry

Immunohistochemistry was performed, as described in more detail recently [11], to detect CD3^+ ^T cells, CD4^+ ^T cells, CD8^+ ^T cells and CD20^+ ^B cells. In brief, sections were immersed in a sodium citrate buffer solution at pH 6.0 and heated in a high-pressure cooker (CD3, CD4, CD8 and CD20). After cooking, the slides were incubated with the respective primary antibodies including monoclonal antibodies against CD4 (clone 1F6; Novocastra, Newcastle, UK), CD34 (QBend10; Immunotech, Marseille, France), and CD3 (F7.2.38), CD8 (C8/144B) and CD20 (L26) obtained from Dako (Glostrup, Denmark). For detection, the alkaline phosphatase/anti-alkaline phosphatase complex (APAAP) method was used, with 'Fast Red' as chromogen.

T-cell and B-cell aggregates were defined as clusters of 50 or more CD3^+ ^T cells or CD20^+ ^B cells, and for each patient the number of CD3^+ ^T-cell aggregates or CD20^+ ^B-cell aggregates in 10 high-power microscopic fields (HPFs), based on a HPF of 0.237 mm^2 ^first, ocular with a 22 mm field of view at ×10 magnification, and a 40× objective), was assessed. For the quantification of interstitial CD4^+ ^and CD8^+ ^T cells and CD20^+ ^B cells in the bone marrow, 10 HPFs were analyzed similarly. The number of interstitial CD4^+ ^and CD8^+ ^T cells was defined as increased if at least ten CD4^+ ^T cells and at least six CD8^+ ^T cells per HPF were present; for CD20^+ ^B cells the criterion was at least five positive cells per HPF on the basis of the findings in zygapophyseal joints of patients without AS, which we used as negative controls.

The results are given semi-quantitatively: +++, at least two CD3^+ ^lymphocytic aggregates and increased interstitial CD4^+^, CD8^+ ^and CD20^+ ^lymphocytic infiltrates; ++, one CD3^+ ^lymphocytic aggregate and increased interstitial CD4^+^, CD8^+ ^and CD20^+ ^lymphocytic infiltrates; +, increased interstitial CD4^+^, CD8^+ ^and CD20^+ ^lymphocytic infiltrates, no aggregates. For the quantification of CD34^+ ^micro-vessel density a semi-quantitative analysis was performed: 0, not more than two micro-vessels per HPF; +, three or four micro-vessels per HPF; ++, five or six micro-vessels per HPF.

### Magnetic resonance imaging

MRI, including T1-weighted, T2-weighted and TIRM (turbo inversion recovery magnitude) sequences, was performed as a preoperative procedure in patients with AS, as shown in Table 1. TIRM sequences allow excellent illustration of the anatomy and detection of inflammation in the bone marrow. The entire lumbar spine including the zygapophyseal joints and, if available, the thoracic spine was analyzed for increased signals in T2-weighted sequences.

## Results

### Mononuclear cell infiltration

We summarize the results of a semi-quantitative analysis of mononuclear cell infiltration in the bone marrow of zygapophyseal joints as described in the Method section. Five patients with AS had the highest score of cellular infiltration (+++) with at least two CD3^+ ^lymphocytic aggregates including increased interstitial CD4^+^, CD8^+ ^and CD20^+ ^lymphocytic infiltrates, and three patients with AS had an increased level of cellular infiltrations (+) without aggregates (Table 1).

### Microvessel density

To address the question of whether hypervascularization might contribute to the detection of bone marrow edema in the spine by MRI we also stained CD34^+ ^endothelial cells in our sections. This issue was examined more thoroughly in a previous paper [10]. The present results are shown in Table 1.

### Bone marrow edema

All patients with AS showed different degrees of interstitial bone marrow edema in histopathological analysis: in two patients with AS, interstitial edema was present in 10% of the total bone marrow area, in two patients it was present in 20%, in two patients in 30% and in two patients in 60% (Table 1). In contrast, none of the controls showed interstitial bone marrow edema.

### Correlation of histopathology with MRI

**Figure 2:**
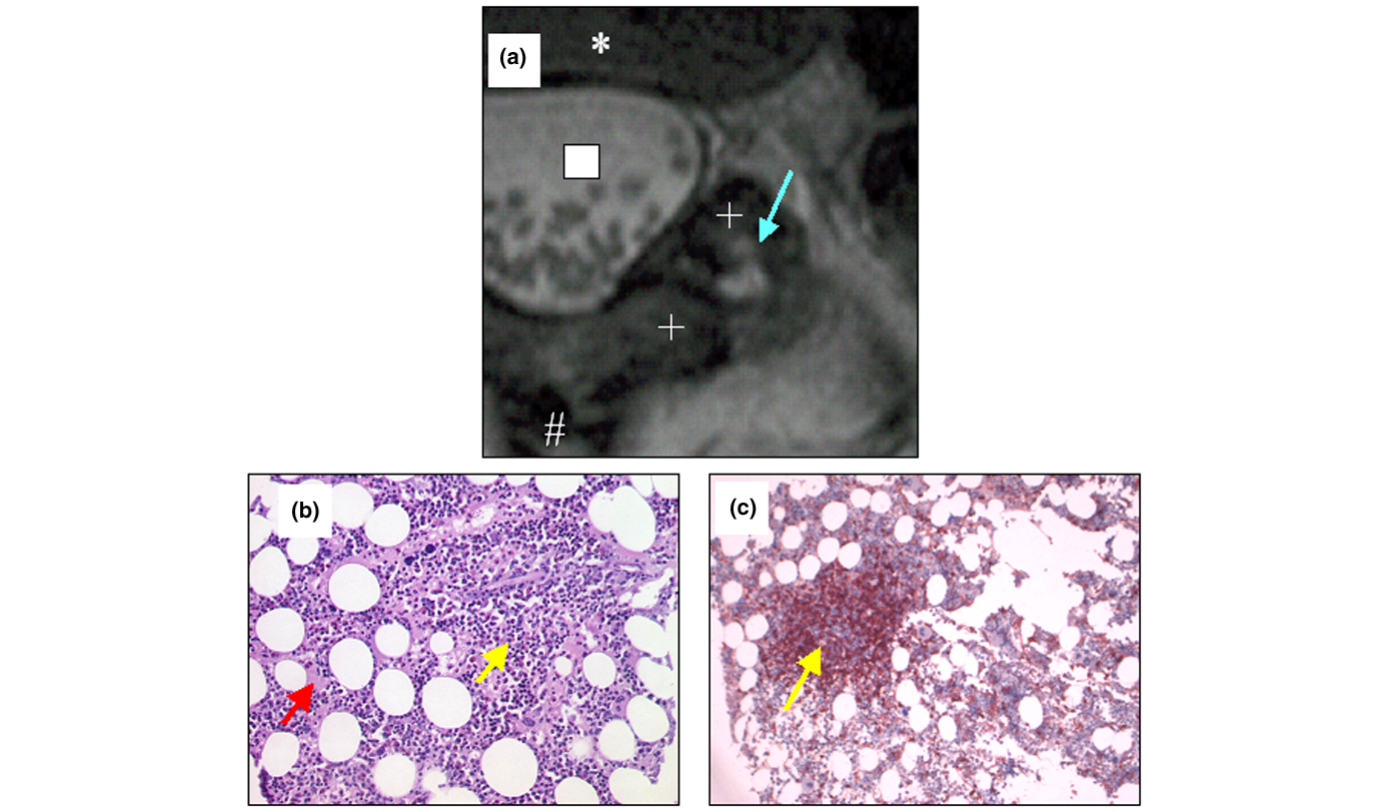
Positive correlation of bone marrow edema in histopathological assessment and magnetic resonance imaging. **(a) **Magnetic resonance imaging (T2 sequence) of a zygapophyseal joint with bone marrow edema (cyan arrow) (asterisk, vertebral body; open square, epidural space; plus signs, superior and inferior articular processes of the zygapophyseal joint; hash sign, processus spinosus). **(b) **Hematoxylin and eosin staining, revealing dense infiltrations of mononuclear cells (yellow arrow) and interstitial bone marrow edema (red arrow). **(c) **Immunohistochemical analysis of CD3^+ ^T cells, revealing mononuclear cell aggregates in the same zygapophyseal joint (yellow arrow).

Interstitial bone marrow edema as detected by histopathology in the zygapophyseal joints of the lumbar spine was found by MRI in three of eight patients with AS (Table 1). Most interestingly, in these patients a good correlation between histopathologically confirmed interstitial edema and cellular infiltration was found. Two patients showed the highest score for interstitial bone marrow edema (60%) and for mononuclear cell infiltrates (+++). An example of such an AS patient is shown in Figure 2: bone marrow edema as detected by MRI (Figure 2a) was correlated with interstitial bone marrow edema as detected by hematoxylin and eosin staining (Figure 2b) and T-cell infiltration (Figure 2c). The third patient with AS (no. 3) with bone marrow edema detected by MRI had 30% interstitial bone marrow edema and the highest score of bone marrow infiltation in the histopathological assessment. Some of the patients had inflammatory lesions in other parts of the spine, as indicated in Table 1.

**Figure 3:**
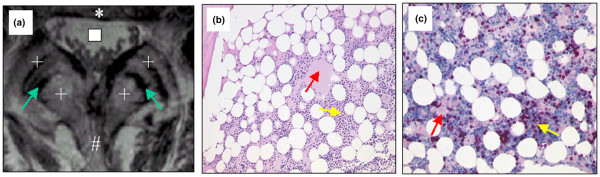
Negative correlation of bone marrow edema in histopathological assessment and magnetic resonance imaging. **(a) **Magnetic resonance imaging of zygapophyseal joints (cyan arrows) without detectable bone marrow edema (asterisk, vertebral body; open square, epidural space; plus signs, superior and inferior articular processes of the zygapophyseal joint; hash sign, processus spinosus). **(b) **Hematoxylin and eosin staining of the zygapophyseal joint, revealing numerous mononuclear cells in the bone marrow (yellow arrow) and a relatively small amount of edema (20%) in the bone marrow (red arrow). **(c) **Immunohistochemical analysis of CD3^+ ^T cells (red arrow), showing dense infiltrates of CD3^+ ^T cells (yellow arrow).

Interestingly, histopathologically detected cellular infiltration and interstitial bone marrow edema was also observed, although to a smaller degree (between 10% and 30%), in patients with negative MRI. An example is shown in Figure 3: interstitial bone marrow edema (20%) (Figure 3b) and cellular infiltration (Figure 3c) was detectable in histopathological observations but not by MRI (Figure 3a). In two of the patients in whom zygapophyseal joints were MRI-negative, signs of osteitis were seen at other sites of the spine: in the vertebral bodies of thoracic vertebrae 11 and 12 of patient 4 and at posterior sites of the vertebral bodies of thoracic vertebrae 6 to 9 including the zygapophyseal joints of patient 6 (Table 1).

## Discussion

Interstitial bone marrow edema in zygapophyseal joints of the lumbar spine as detected by histological examination was present in all eight patients with AS to a different degree, varying from 10% to 60% of the total bone marrow area. There was a clear correlation between histopathologically confirmed interstitial edema and edema as shown by MRI. However, a small amount of histopathological interstitial edema in the bone marrow (less than 30% of the surface area) is not detected by MRI. There was only a poor correlation between histopathologically observed interstitial edema and inflammatory cell infiltration, and therefore only a poor correlation between cell infiltration and MRI edema, which might explain why some patients with AS can have active disease despite a normal MRI [16]. An earlier study comparing AS histology from computed-tomography-guided biopsies from the sacroiliac joint with MRI observed some correlation between cell infiltration and bone marrow edema by MRI but did not investigate and compare histopathological and MRI bone marrow edema [9].

We are aware that this analysis has its limitations: the number of patients in this study is small, all patients had advanced disease progression, the slices of MR images were 4 mm thick or were for example available only in sagittal sections (patients 7 and 8) and because of that it might be possible that inflammation and edema was missed. Furthermore, transverse images of the zygapophyseal joints might be more sensitive [8]. However, the protocol used for MR images is a standard protocol for daily routine and should therefore be used for comparative analysis.

In patients with AS, spinal MRI is being used to assess spinal inflammation as an indicator of disease activity. Lesions of active inflammation are depicted as areas of increased signal intensity in T2-weighted images with fat saturation (STIR sequences) and most probably represent an increased water content, probably as a correlate of bone marrow edema [17]. The term 'bone marrow edema' in MR images was first used by Wilson and colleagues in 1988. Regional decreased signal intensity of the bone marrow in T1-weighted images and increased signal intensity on T2-weighted images represented an accumulation of 'bone marrow water', which could be confirmed by biopsy. They defined such lesions as 'bone marrow edema' [18]. A first systematic analysis of bone marrow edema in MR images and histopathological analysis was performed in osteoarthritis [15] in which bone marrow edema could be observed in MR images in up to 50 to 68% of patients. In this study the bone marrow edema, defined as a hyperintense zone on STIR images and hypointense on T1-weighted MR images, consisted mainly of normal tissue (53% of the area was fatty marrow, 16% was intact trabeculae, and 2% was blood vessels) and in a smaller proportion of other changes, namely interstitial bone marrow edema 4% [15]. Taken as a whole, the study by Zanetti and colleagues [15] revealed non-characteristic histopathological abnormalities without increased infiltrations of mononuclear cells, increased microvessel density or interstitial bone marrow edema, clearly indicating that MRI-detected 'bone marrow edema' in patients with osteoarthritis can have various underlying histomorphological alterations. In our study the presence of large numbers of cellular infiltrates in all patients with a greater percentage of histopathologically confirmed interstitial edema argues strongly for the hypothesis that the bone marrow edema in MRI in our cohort of patients with AS was caused by inflammation.

## Conclusion

There is a good correlation between histological and MRI edema in patients with AS, although MRI seems to be less sensitive than histopathological analysis. Edema is a product of inflammatory reaction but our histopathological observations indicate that inflammation reflected by cellular infiltrates does not always cause the same degree of edema. This might be an explanation for the sometimes observed discrepancy between high disease activity and negative MRI in AS. Further investigations comparing radiological and histological changes will be needed to clarify this topic. Therefore, other structures involved in AS, for example hip joints and knees obtained from joint replacement, should be analyzed in future.

## Abbreviations

AS = ankylosing spondylitis; HPF = high-power field; MR = magnetic resonance; MRI = magnetic resonance imaging; TIRM = turbo inversion recovery magnitude.

## Competing interests

The authors declare that they have no competing interests.

## Authors' contributions

HA, CL and JS were responsible for study design, manuscript preparation, data acquisition and interpretation, and statistics. ZG and MR were responsible for acquisition of MRI data and its interpretation, and for manuscript preparation. HE was responsible for data acquisition from patients without AS and for interpretation of data. MD, AH and PM-S were responsible for data acquisition from patients with AS and for interpretation of data. HS was responsible for data acquisition and interpretation and for manuscript preparation. All authors read and approved the final manuscript.

## Figures and Tables

**Table 1:** Correlation of histopathological findings and magnetic resonance imaging

Patient no.	HLA-B27	Edema (%)	Cellular infiltration	Microvessel density	Edema detected by magnetic resonance imaging	
					
					Zygapophyseal joints of lumbar spine	Other segments of lumbar or thoracic spine
1	-	60	+++	++	L1, L2	Th12
2	+	60	+++	+	L2, L3	-
3	-	30	+++	+	L2, L3, L4	Th5–Th7 and zygapophyseal joints Th5–Th7
4	+	20	+++	+	-	Thoracic spine Th11, Th12
5	+	10	+	+	-	-
6	+	20	+	+	-	Th6–Th9 and zygapophyseal joints Th6–Th9
7	+	30	+++	+	-	-
8	+	10	+	+	-	-

L, vertebra of the lumbar spine; Th, vertebra of the thoracic spine.
